# Confocal fluorescence resonance energy transfer microscopy study of protein-protein interactions of lens crystallins in living cells

**Published:** 2007-06-14

**Authors:** Bing-Fen Liu, Kumarasamy Anbarasu, Jack J-N. Liang

**Affiliations:** 1 Ophthalmic Research/Surgery, Brigham and Women's Hospital, Boston, MA; 2 Department of Ophthalmology, Harvard Medical School, Boston, MA

## Abstract

**Purpose:**

To determine protein-protein interactions among lens crystallins in living cells.

**Methods:**

Fluorescence resonance energy transfer (FRET) microscopy was used to visualize interactions in living cells directly. Two genes, one (αA-crystallin) fused with green fluorescence protein (GFP) and the other (each of the following genes: αB-, βB2-, γC-crystallin, and R120G αB-crystallin mutant) fused with GFP variant red fluorescence protein (RED), were cotransfected into HeLa cells. After culture, confocal microscopy images were taken and FRET values were calculated.

**Results:**

FRET occurs when the two proteins interact. The data show strong interactions between αA- and αB-crystallin and weak interactions between αA- and βB2- or γC-crystallin, which is consistent with our previous two-hybrid system study. The R120G αB-crystallin mutant, however, showed significantly less FRET than wild-type αB-crystallin. There are also more R120G αB-crystallin transfected cells with protein aggregates than wild-type αB-crystallin transfected cells. Cotransfection with αA-crystallin could not rescue R120G αB-crystallin from aggregation.

**Conclusions:**

FRET microscopy gave excellent results on the protein-protein interactions among crystallins. It supports many previous studies and provides a novel technique for further study of protein-protein interactions among lens proteins including membrane and cytoskeletal proteins.

## Introduction

Protein-protein interaction plays an important role in cellular and protein functions. Crystallins, the major protein components in the lenses, are grouped into two families: α- and βγ-crystallins [1]. While α-crystallin is composed of only two polypeptide subunits, αA- and αB-crystallin, forming a hetero-oligomer, βγ-crystallins are more heterogeneous with seven subunits (four acidic βA1-, βA2-, βA3-, and βA4-crystallin, and three basic βB1-, βB2-, and βB3-crystallin) in the heterooligomeric β-crystallin and six subunits (γA-γF-crystallin) in the monomeric γ-crystallin. In vitro, αA- and αB-crystallin can also form homo-oligomers. One inevitable question arises: why do the lenses need so many different crystallins? The answer may lie in the fact that interactions among the crystallins stabilize the protein structures. The nature of interaction in highly concentrated solutions or in the lens is a short-range order of crystallins that accounts for the lens transparency [2]. If this protein interaction is disrupted, refractive index gradients are compromised, which leads to light scattering and cataract formation [3]. Further studies in the protein solutions or in the cells indicate that not only the presence but also the extent of protein-protein interaction can be evaluated. Many methods have been used in studying protein-protein interactions among crystallins in solutions including light scattering [4], fluorescence polarization [5], fluorescence resonance energy transfer (FRET) [6,7], surface plasmon resonance [8], microequilibrium dialysis [9], peptide scans [10], and protein pin array [11]. These studies were performed with protein solutions requiring extensive protein purification. However, studying protein-protein interactions in the cells with a two-hybrid system removes the necessity of protein purification [12,13]. Earlier research in lens proteins focused on posttranslational modifications associated with age-related cataract (ARC); recently, the focus was shifted to site-specific mutations seen in some congenital cataracts. Both posttranslational modifications and mutations must disrupt protein-protein interactions. We have reported changes of protein-protein interactions due to mutations using the two-hybrid system assay [14,15]. However, the two-hybrid system has the possible disadvantage that the two expressed proteins need to move to the nucleus for transcriptional activation of the reporter gene; a large protein, such as α-crystallin or a membrane protein, may have difficulty entering the nucleus. In the present study, we implemented confocal FRET microscopy to detect protein-protein interactions between αA-crystallin and other crystallins, using green fluorescence protein (GFP) and its variant, red fluorescence protein (DsRED). We prepared the first fusion protein of GFP-αA-crystallin as the donor and the second fusion protein of DsRED-other crystallins as acceptor. FRET microscopy provides an advantage by allowing direct assessment of protein-protein interactions in the natural environment in living cells [16]. Unlike test tube experiments, the method requires no protein purification and contrary to the two hybrid system, it does not need a reporter gene or cell lysis. The FRET images allow us to directly visualize the protein-protein interactions and also to obtain quantitatively the extent of interactions.

## Methods

### Plasmid Construction of GFP and DsRED with crystallins

The pAcGFP-C1 and pDsRED Monomer-C1 vectors were purchased from Clontech (Palo Alto, CA). The pAcGFP1-C1 vector is encoded with a green fluorescent protein (GFP) from *Aequorea coerulescens* (λ_ex_/λ_em_=475/505 nm). The pDsRED-Monomer-C1 is encoded with a DsRED-Monomer (DsRED.M1) with a red fluorescent protein DsRED, a mutant derived from the tetrameric *Discosoma* sp (λ_ex_/λ_em_=557/585 nm). The αA-, αB-, βB2-, and γC-crystallin, as well as R120G αB-crystallin genes (pM-αA-, pM-αB-, pM-βB2-, pM-γC-, and pM-R120GαB-crystallin) were prepared in our previous studies [12,14]. The αA gene was subcloned into pAcGFP-C1 and the other crystallin genes were subcloned to pDsRED monomer-C1 terminals. The resulting constructs were designated as GFP-αA-, RED-αA-, RED-αB-, RED-R120GαB-, RED-βB2-, and RED-γC-crystallin. The PCR was performed using the forward primers containing the *xho*I restriction site and reverse primers containing the *Eco*RI site (Table 1). The primers were custom synthesized (Invitrogen, Carlsbad, CA). All constructs were verified by Sanger sequencing with an ABI Automatic Sequencing System (Perkin Elmer Applied Biosystems Inc., Foster City, CA) at Brigham and Women's Hospital core facility.

**Table 1:** Primers for subcloning crystallins to GFP and RED vectors.

	**Forward primers**	**Reverse primers**
αA	GACCCTCGAGCTGACGTGACCATCCAG	AGCCTAGAATTCTTAGGACGAGGGAGC
αB	AACCCTCGAGCTGACATCGCCATCCAC	CAGGAGGAATTCCTATTTCTTGGGGGC
βB2	GAACCTCGAGCTGCCTCAGATCACCAG	GGTCTAGAATTCCTAGTTGGAGGGGTG
γC	TAGCCTCGAGCTGGGAAGATCACCTTC	CGCCGCGAATTCTTAATACAAATCCAC
R120G αB	AACCCTCGAGCTGACATCGCCATCCAC	CAGGAGGAATTCCTATTTCTTGGGGGC
G-17-R	AAAGGTCCCCGGTCGCCACCATGGACA	TTTGGATCCCTGGGAGCCGGAG

The forward and reverse primers for subcloning experiments of various crystallins to pAcGFP-C1 and pDsRED-monomer-c1 vectors. The underlined sequences are for *xho*I and *Eco*RI restriction sites for 5' and 3' primers for crystallins, and *Kpn*I and *Bam*HI for G-17-R, respectively.

A construct of GFP-RED fusion protein was prepared for use as a positive control by subcloning RED cDNA from pDsRED Monomer-C1 to pAcGFP-C1 using *Kpn*I/*Bam*HI restriction site (Table 1). The linker length between GFP and RED is 17 amino acids and the fused protein was named G-17-R.

### Cotransfection and Cell Culture

HeLa cells were cultured in an MEM medium supplemented with 10% fetal calf serum, 2 mM glutamine, and 1% penicillin/streptomycin antibiotics (GIBCO BRL, Gaithersburg, MD) in a 5% humidified CO_2_ incubator at 37 °C. One day before transfection, HeLa cells (1x10^5^/ml) were seeded into a 35 mm culture dish and cotransfected with vectors using the lipofactamine 2000 reagent at the ratio 1:2 (1.6 μg of each cDNA: 3.2 μg of lipofactamine 2000). After incubation for six h, the medium was changed and incubation continued up to 48 h for imaging analysis. Prior to the imaging experiments, the culture medium was replaced with L-15 medium without FCS.

### Fluorescence resonance energy transfer analysis with confocal fluorescence microscopy

Images were acquired with a Zeiss Laser Scanning Microscope (LSM) 510 META Axioplan 2 confocal microscope (Carl Zeiss, Inc., Thornwood, NY) at the Harvard Center for Neurodegeneration & Repair, Optical Imaging Facility. Images (12-bit) of multitrack channels with the following configuration were recorded: an argon/2 laser (25 mW, T1 and T3=10% of laser exposure) for the pAcGFP channel (donor excitation/ donor emission: GFP_ex/em_) and FRET channel (donor excitation/ acceptor emission: FRET_ex/em_) with excitation wavelength at 488 nm and a HeNe 1 Laser (T2=100%) for the pDsRED channel (acceptor excitation/acceptor emission: RED_ex/em_) with excitation wavelength at 543 nm.

LSM images were analyzed with the automated Metamorph 7.0 software (Molecular Devices, Sunnyvale, CA). The intensities of total gray values in the three channels (I_GFP,_ I_RED,_ and I_FRET_) were measured in the same region of interest (ROI) by Integrated Morphometry Analysis. In order to obtain the true FRET signal, the net FRET (I_nFRET_) threshold-adjusted images were plotted to an Automated Application program and corrected for contribution from spectral bleed-through (SBT) of the donor and acceptor fluorescence emission in the FRET signal:

InFRET=IFRET−IREDx⁡a−IGFPx⁡b

where *a* is the coefficient due to SBT of acceptor signal to FRET channel and *b* is the coefficient due to SBT of donor signal to FRET channel and were determined with cells expressing DsRED construct only (with RED signal but no GFP signal) or AcGFP construct only (with GFP signal but no RED signal) and were defined by the following equations:

a=FRETex/em/REDex/em

and

b=FRETex/em/GFPex/em

The contributions from SBT are removed by the computer program. In order to make the values of I_nFRET_ comparable among different ROIs and cells, I_nFRET_ was normalized with I_GFP_ intensity.

### Statistical analysis

Data are expressed as the mean±SE from a minimum of three independent experiments. Statistical analysis was performed with Student's *t*-test using Sigmaplot statistical analysis software (Systat Software, San Jose, CA) with a p<0.05 as the criterion of significance.

## Results

### Transfection of individual constructs

LSM images of HeLa cells transfected with each construct indicate that while the distribution of αA- and αB-crystallin is confined mostly in the cytoplasm, βB2- and γC-crystallin were found in both the cytoplasm and the nucleus. The result suggests that αA- and αB-crystallin had difficulty entering the nucleus possibly because of their large size.

The SBT coefficients were estimated that GFP channel contributes about 35% to the FRET channel and the RED channel contributes about 5%. This SBT contamination is the major problem in FRET microscopy. We chose the GFP-RED pair, which shows much less SBT than many other frequently used pairs, such as the CFP-YEP pair, which reportedly 70% of the cyan channel signal and 20% of the yellow channel signal cross over to the FRET channel [17].

### Cotransfection of GFP-αA and RED-crystallin constructs

**Figure 1:**
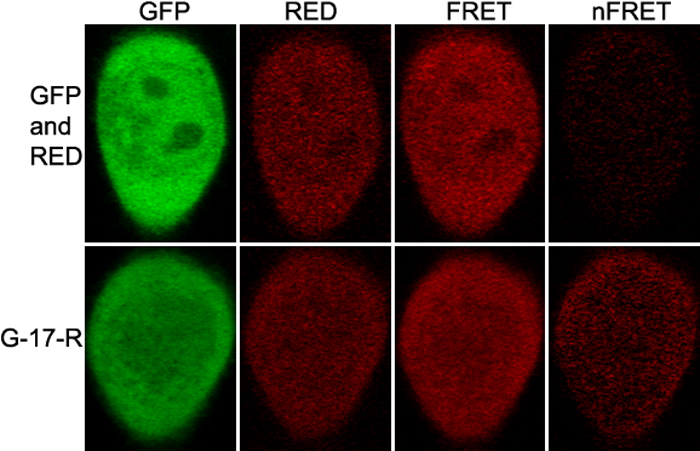
Laser scanning microscope fluorescence resonance energy transfer images of cells expressing either the control G-17-R or negative control. Shown on the top of the columns are various channels and normalized FRET and on the left are RED acceptors. Either the G-17-R construct or the paired constructs (GFP and RED) were transfected to HeLa cells. After culture, laser scanning microscope FRET images were acquired. The images show that normalized FRET is great for G-17-R transfected cells but very faint for (GFP and RED) cotransfected cells.

Initially, we performed control experiments using G-17-R fusion protein as the positive control and individual GFP and RED genes as the negative control. When G-17-R was transfected into HeLa cells, a strong FRET signal was observed. however, when GFP and RED were cotransfected in the HeLa cells, a very faint signal was observed (Figure 1). These observations strongly suggested the feasibility of using fusion GFP and RED proteins in our study as probes to detect protein-protein interactions.

**Figure 2:**
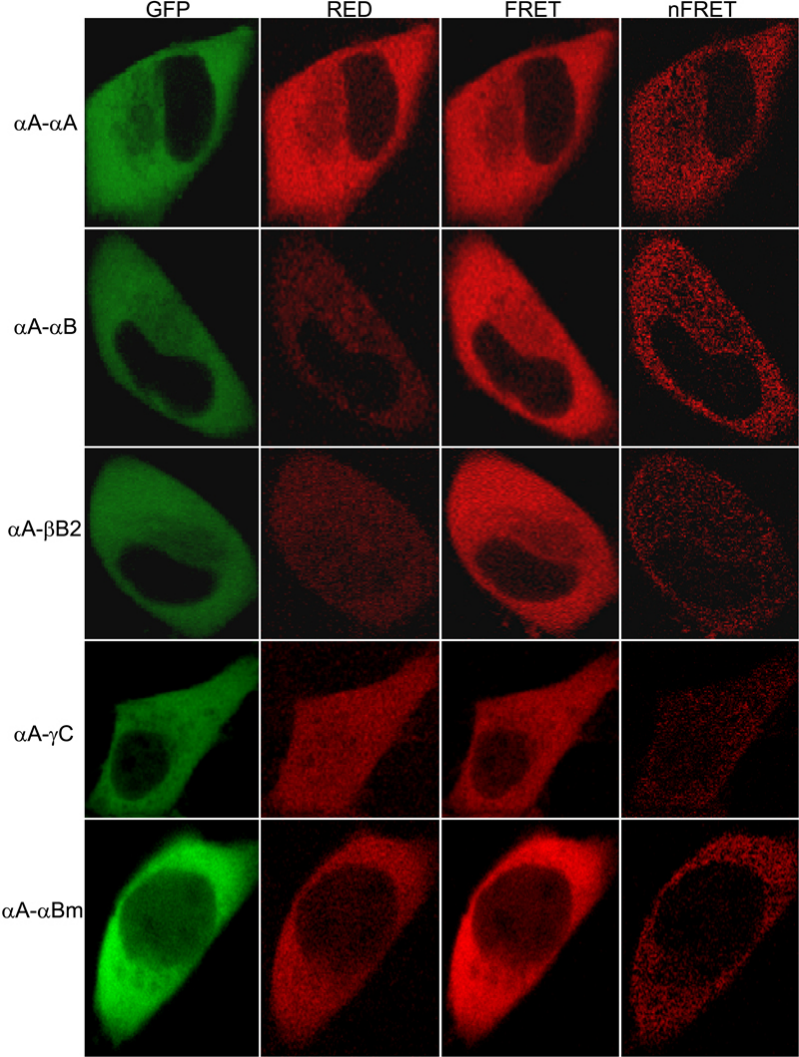
Representative laser scanning microscope fluorescence resonance energy transfer images of cells expressing the GFP and RED constructs. GFP-αA-crystallin as a donor and RED-crystallin (αA-, αB-, βB2-, γC-, and R120G αB-crystallin) as an acceptor. Shown on the top of the columns are various channels and normalized FRET and on the left are GFP-crystallin (donor) and RED-crystallin (acceptor) pairs. Paired constructs were cotransfected to HeLa cells. After culture, laser scanning microscope FRET images were taken. The data show that FRET is greater between αA- and αB-crystallin than between αA- and βB2- or γC-crystallin.

**Figure 3:**
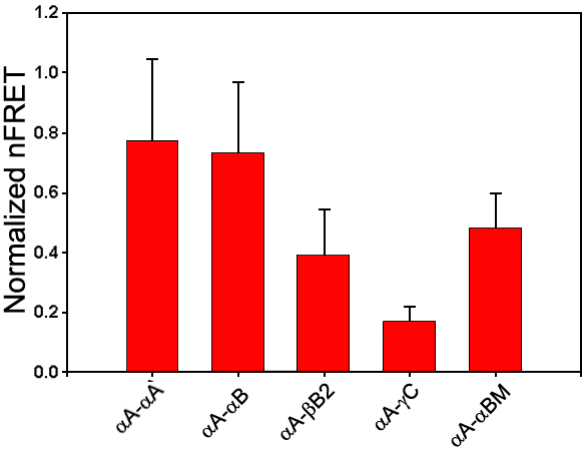
The quantitative normalized fluorescence resonance energy transfer values of crystallins were calculated from laser scanning microscope fluorescence resonance energy transfer images. The nFRET values are the average of regions of interests (ROIs; n=60-90) in three transfection experiments. ROIs were chosen in the areas that contain no protein aggregates. Significant differences were observed for the pairs (αA- or αB- and βB2-crystallin), (αA- or αB- and γC-crystallin), (βB2- and γC-crystallin), and (αB- and R120G αB-crystallin. The asterisk indicates a p<0.001), but insignificant for the pair (αA- and αB-crystallin; p=0.2).

We used GFP-αA-crystallin as the donor and each of the other crystallins (RED-crystallin) as the acceptor in our FRET microscopy experiments. The results reveal that all other crystallins interact with αA-crystallin (Figure 2). When analyzed for the quantitative nFRET/GFP values, the different extent of interactions between αA-crystallin and other crystallins becomes obvious (Figure 3). The order is αA-crystallin~αB->βB2->γC-crystallin. The difference between αA- and αB-crystallin is statistically insignificant. The significant decrease of normalized nFRET for R120G αB-crystallin from WT αB-crystallin is also obvious.

**Figure 4:**
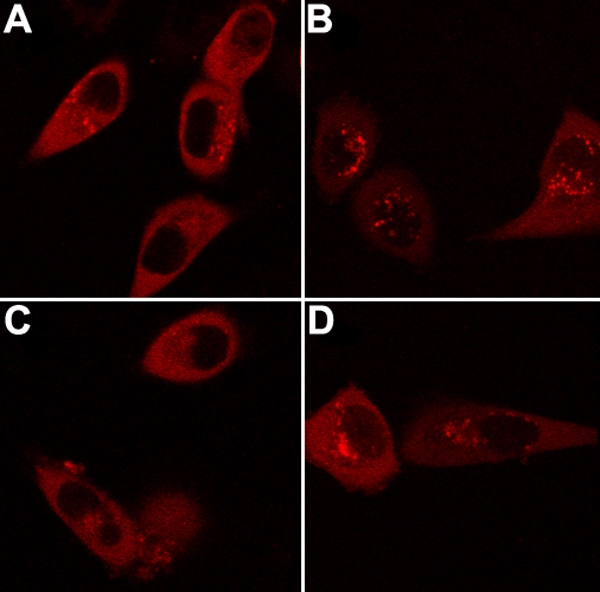
Representative laser scanning microscopy images of cells transfected or cotransfected with αA- and αB-crystallin genes. **A**: RED-αB-, **B**: RED-αBM-, **C**: GFP-αA- and RED-αB-, and **D**: GFP-αA- and RED-αBM-crystallin. Only images of the RED channel were shown. Constructs were transfected to HeLa cells. After culture, laser scanning microscope images were taken. The data show that cells transfected with αBM-crystallin were more susceptible to form protein aggregates than cells transfected with WT αB-crystallin and cotransfection with αA-crystallin could not reduce cells of protein aggregates for αBM-crystallin as much as for WT αB-crystallin.

**Figure 5:**
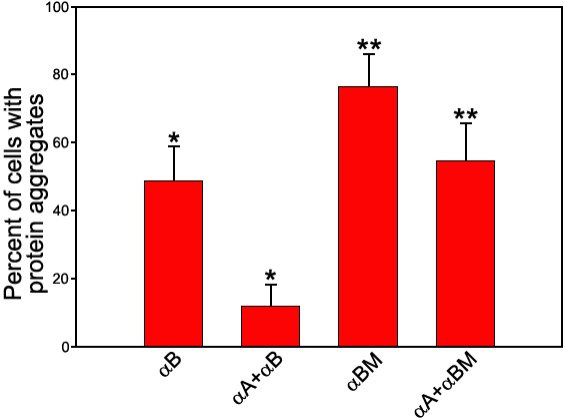
The percentage of cells with protein aggregates cotransfected with αA- and αB-crystallin or R120G αB-crystallin (αBM) genes. The x-axis labels are either RED-αB- or the pairs of GFP-αA- and RED-αB-crystallin. A four-fold decrease of cells with protein aggregates was observed for cotransfection of αA- and αB-crystallin (the asterisk indicates a p=0.006), but only about a 1.5 fold decrease for cotransfection of αA- and αBM-crystallin (the double asterisk indicates a p=0.02). The mean and standard deviation are shown as percentage ±SD and represent an average of three independent experiments. For each experiment, cells were counted in 30-50 confocal LSM images.

There are protein aggregates in the cells transfected with αB-crystallin. Cotransfection of the αA- and αB-crystallin genes decreased the aggregation greatly; the percentage of cells with protein aggregates decreased more than fourfold from the αA- and αB-crystallin transfected cells (Figure 4 and Figure 5). Cells with protein aggregates were counted regardless of the size or number of protein aggregates in all LSM pictures of each experiment. In fact, we observed not only greater number of cells with protein aggregates but also larger protein aggregates in the cells transfected with R120G αB-crystallin than in the cells transfected with WT α-crystallin. It is to be noted that there are hardly any protein aggregates in the cells transfected with αA-crystallin alone (data not shown). Thus, the interaction between αA- and αB-crystallin increased αB-crystallin solubility greatly. However, the congenital cataract mutant R120G αB-crystallin behaved quite differently; its aggregation was greater than the wild-type αB-crystallin. The percentage of cells with protein aggregates decreased only 1.5 fold from the αA- and R120G αB-crystallin transfected cells, indicating that αA-crystallin could not rescue R120G αB-crystallin from forming protein aggregates as much as the wild-type αB-crystallin.

## Discussion

Studying protein-protein interaction using color GFP by FRET in living cells is one of the recent advances in protein biology. In FRET, the energy is transferred nonradiatively from the donor protein to the acceptor protein when they are expressed in very close proximity (about 50 Å) and when the emission spectrum of the acceptor protein overlaps with the excitation spectrum of the donor protein [18]. In vitro experiments with FRET have been extensively used to detect protein-protein interactions with either two extrinsic fluorescent probes or one intrinsic probe (Trp) and one extrinsic probe. In crystallin research, most studies focus on FRET detection of subunit exchange and its change due to mutations [19-23]. In vitro studies suffer from one disadvantage, they require extensive purification processes, and the conformation of purified protein may differ from the in vivo environment. Our previous study using the two-hybrid system detected protein-protein interactions in the cells and eliminated the need for protein purification [12]. Both the two-hybrid system and FRET microscopy provide this advantage but the latter technique probes protein-protein interaction inside living cells and should be more physiologically relevant. Another advantage of microscopy FRET is the two expressed proteins do not require relocating to the nucleus for interaction to activate the reporter gene transcription as is required in the two-hybrid system. In spite of possessing the nuclear relocation sequence, a large protein or membrane protein may have difficulty moving to the nucleus.

The detection of protein-protein interactions by FRET microscopy among crystallins (αA-αA-, αA-αB-, αA-βB2-, and αA-γC-crystallin) is consistent with the report of a two-hybrid system study in the HeLa cells [12] and of a FRET study in protein solutions [7]. Although subunit exchange may be involved in αA- and αB-crystallin, the nature of observed interactions detected by both the two-hybrid system and FRET microscopy is not known. Hydrophobic interactions may also have contributed to the increased FRET since αA- and αB-crystallin have much greater hydrophobicity than βB2- and γC-crystallin [24]. Protein-protein interactions play an important role in stabilization of protein structure. One example is based on our recent findings that co-expression of βB2- and βA2-crystallins greatly enhanced solubility of βA2-crystallin, which is not soluble when expressed alone (unpublished). Previous reports of βA4-βB2 and αA-αB also show similar effects [25,26]. Posttranslational modifications or site-specific mutations will disrupt or decrease the interactions. FRET study of these changes in protein-protein interactions has been widely reported in protein solutions [21,27-30]. We have reported of these changes in the cells by site-specific mutations with the two-hybrid system assay [14,15]. It is possible to extend the present studies to some posttranslational modifications such as deamidation and COOH-terminal truncation, which can be manipulated by site-specific mutations. Deamidation has been reported to be a major posttranslational modification in aged human lenses [31] and it leads to destabilized proteins [32,33]. COOH-terminal truncations have also been observed in aged and diabetic lenses [34,35] and decreased subunit exchange for 5AA-truncated αA-crystallin was reported [36]. Both deamidation and COOH-terminal truncation must have changed protein structures and interaction properties.

Many congenital cataract crystallin genes have been reported; the R120G αB-crystallin causes desmin-related myopathy and cataract [37]. Recombinant R120G αB-crystallin has been reported to have an irregular structure, decreased chaperone-like activity [38], and decreased protein-protein interactions with αA-crystallin [14]. Decreased nFRET for R120G αB-crystallin in the present study is consistent with those observed in protein solutions [7] and in the cells [14]. Our confocal microscopy images further indicate that the R120G αB-crystallin is far less soluble than the wild-type αB-crystallin and cotransfection with αA-crystallin could not rescue it from aggregation as much as the wild-type αB-crystallin. The results imply that the formation of protein aggregates for the R120G αB-crystallin is the main mechanism for lens opacity.

The significance of protein-protein interactions is to form protein complexes for cellular functions. In the lenses, crystallins interact to generate a gradient of refractive index so that light can focus to the retina with minimal scattering [3]. Another role of protein interactions is to enhance protein stability or solubility. With no turnover, lens proteins have suffered from age-related posttranslational modifications, which destabilize protein structure. In order to maintain the normal protein and lens structures, various crystallins need to interact with one another to strengthen their structural stability. We believe our report to be the first to undertake FRET microscopy study on crystallins. The results not only confirm our previous results in a two-hybrid system but also introduce a better technique for future study of the protein-protein interactions among lens proteins, for example, between crystallins and membrane proteins or cytoskeletal proteins.
